# Two Birds with One Stone: Health Care Providers’ Perspectives about Prevention Technologies in Kenya and South Africa

**DOI:** 10.1177/2325958219841366

**Published:** 2019-04-25

**Authors:** Alexandra Lutnick, Mary Kate Shapley-Quinn, Kgahlisho Nozibele Manenzhe, Jacob Onyango, Kawango Agot, Khatija Ahmed, Ariane van der Straten

**Affiliations:** 1 RTI International, Behavioral and Urban Health Program, San Francisco, CA, USA; 2 RTI International, Women’s Global Health Imperative (WGHI), San Francisco, CA, USA; 3 Setshaba Research Centre, Soshanguve, South Africa; 4 Impact Research And Development Organisation, Kisumu, Kenya

**Keywords:** HIV, unintended pregnancy, multipurpose technologies, South Africa, Kenya, qualitative

## Abstract

To meet the reproductive health needs of women, especially those in sub-Saharan Africa, multipurpose prevention technologies (MPTs) that combine pregnancy and HIV prevention into a single product could be highly beneficial. This qualitative study with health care providers in Kenya and South Africa examined health system factors that may facilitate or inhibit the delivery of these MPTs. Twelve qualitative interviews were conducted with health care providers at each site (24 interviews total). Providers were presented with pictures and actual placebo prototypes of 4 MPTs: a vaginal ring, an oral pill, an injectable, and an implant. Four themes emerged related to health care providers’ reported interest in offering the proposed MPTs: (1) perceptions of young women’s interest in the MPTs, (2) considerations about product administration, (3) feedback about product attributes, and (4) providers’ training needs. Overwhelmingly, health care providers are eager to offer a product that prevents both HIV and unintended pregnancy in young women.

**What Do We Already Know about This Topic?**

As new products are developed that could simultaneously prevent unintended pregnancy and HIV, the insight of health care providers is needed as they will be key gatekeepers in providing education and access to these products.

**How Does Your Research Contribute to the Field?**

The TRIO study conducted in-depth interviews with health care providers in South Africa and Kenya to understand their perspectives on women’s interest in 3 multipurpose prevention products, considerations for product administration, feedback about product attributes, and training needs.

**What Are Your Research’s Implications toward Theory, Practice, or Policy?**

These results indicate that health care providers in South Africa and Kenya are enthusiastic about the possibility of providing MPTs to their clients but are conscious of the resource limitations that could be obstacles such as: sufficient staff, time for adequate training, and supply chain issues.

## Introduction

Worldwide, Sub-Saharan Africa has the greatest percentage of women (58%) who are considered “in need of contraception”^1^ and is also the region that bears the greatest burden of HIV. Coverage with clinically recommended contraceptive methods (ie, injectables, pills, condoms, intrauterine devices (IUDs), and implants) is insufficient, as it is estimated that unintended pregnancies would decrease by 83% if sub-Saharan African women’s need for contraception was met.^2^ Likewise, women in this region, particularly young women, are disproportionately impacted by HIV.^3^ Women acquire HIV 5 to 7 years earlier than men, and those who are 15 to 24 years old are twice as likely to have HIV as their male counterparts.^4^ This imbalance is rooted in a complex combination of factors, including biological susceptibility, sexual behavior norms, and gendered social norms.^5,6^ Condom use remains problematically low,^7^ despite its proven effectiveness as a method of contraception and HIV prevention.^8^ Barriers to condom use among young women are well noted (lack of availability, perceptions of decreased pleasure, desire or pressure to become pregnant, and challenges negotiating their use).^9^

Preventing both unintended pregnancies and HIV is a dual health priority for women in sub-Saharan Africa. Recognizing the need for more options that can simultaneously prevent HIV and unintended pregnancy, researchers have made progress toward developing new multipurpose prevention technologies (MPTs). As several types of products are moving into the development pipeline, researchers and advocates have highlighted the importance of evaluating the acceptability of these potential MPTs among the end-user population most in need: young women in sub-Saharan Africa. Health care providers’ perspectives are also needed because they will be the ones providing education and access to these products.^10-16^ An MPT that combines both pregnancy and HIV prevention would provide women with a single empowering tool that protects against multiple risks. Such products may increase the acceptability, uptake, and adherence of prevention methods by minimizing burden, simplifying use, and capitalizing on the opportunity to dovetail disease prevention with the less stigmatized indication of contraception.^15^

The Trio study, conducted in Kenya and South Africa, contributes to efforts for developing MPTs that would be acceptable to young women.^17-19^ Within the broad range of contextual, social, and individual factors that may influence women’s acceptability and use of an MPT, the focus in this article is on the health system factors that may facilitate or inhibit the delivery of these MPTs. In-depth interviews (IDIs) with health care providers offered insight into whether they think young women will want to use these products, considerations about product administration, feedback about product attributes, and details about the providers’ training needs.

## Methods

As part of the Trio Study, research staff at Setshaba Research Center (SRC) in Soshanguve, South Africa, and at Impact Research & Development Organization (IRDO) in Kisumu, Kenya, conducted IDIs with health care providers. Both SRC and IRDO are nongovernmental organizations (NGOs) that implement HIV prevention research and programs.

Each site interviewed 12 key informants who worked in a variety of health care service delivery settings such as nurses, counselors, and doctors for a total of 24 interviews. Key informants were purposively recruited through sites’ known networks of health care providers in their catchment areas. In selecting health care providers, sites aimed to assemble a diverse sample of influential stakeholders representing various roles and sectors in the health care system, with a focus on those who directly provide services to young women. Health care providers came from a range of service settings. In South Africa, the sample included providers from a youth center, local clinics, a district health office, a research center, and hospitals. In Kenya, the sample included providers from private and public hospitals, drop-in clinics, NGOs, and facilities specializing in HIV programming.

**Figure 1.**
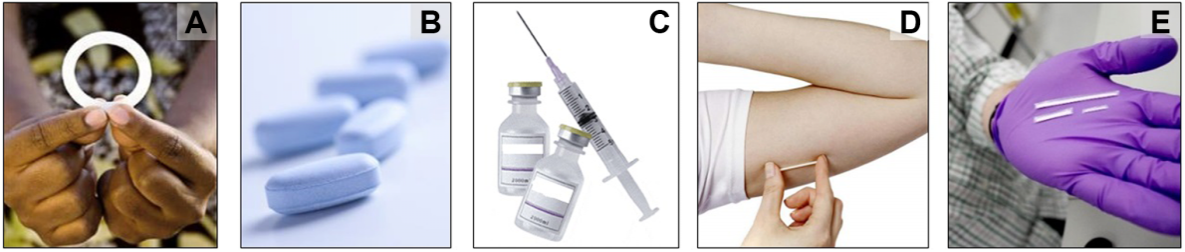
Hypothetical multipurpose technology (MPT) products presented to health care providers. (A) vaginal ring, (B) pill, (C) injection, (D) implant, and (E) biodegradable implant.

**Figure 2.**
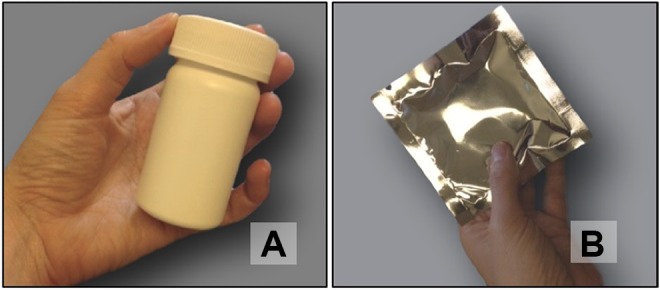
Hypothetical multipurpose technology (MPT) product packaging presented to health care providers. (A) vaginal ring; (B) pill.

Data were collected using a semi-structured interview guide, and all interviews were conducted in English by social science staff who had received standardized study training as well as specific training on qualitative data collection. Ongoing quality control mechanisms were in place to ensure all interviewers met expectations for interviewing skills. For each key informant, demographic data were also collected. In-depth interviews explored service delivery factors that would influence young women’s uptake of MPTs and sought to understand health care providers’ attitudes about these MPTs (Table 1). During the interviews, informants were presented with pictures (Figure 1) and actual placebo prototypes of the 3 TRIO products: a vaginal ring used for 1 month, a daily oral pill, and a monthly injectable (2 injections, 1 in each gluteal muscle). They were also presented with a fourth product, an implant (both biodegradable and nonbiodegradable), that provides medication for 3 to 6 months.^20^ Key informants were also shown prototypes of product packaging for the vaginal ring and the daily oral pills (Figure 2). Finally, key informants were prompted to assume that all products offered equal efficacy.

**Table 1.** General Topics and Example Questions in the Interview Guide for Health Care Provider Interviews.

General Topic	Example Questions
Background	What is your role in the community? What type of HIV prevention and/or family planning work do you do with young people, aged 18-30?
HIV	What types of methods or behaviors do young women use most frequently to protect themselves against HIV? Please list all the products and methods you are aware of that may protect against HIV infection
Family planning	What types of family planning methods do you promote in your work with young women? What impacts a young woman’s ability to properly use each kind of family planning method?
Trio products	What kinds of things would make you interested in providing or promoting each of these products to young women? Please discuss the workflow process in the clinic that would be involved with providing these products

On average, the interviews lasted 83 minutes. The interviews were digitally recorded and transcribed verbatim. The transcribed IDIs were imported into NVivo 11 Pro^21^ for coding and analysis. Interviews were conducted between August and December 2015.

A template approach,^22^ coupled with thematic analysis, was used to analyze the data. The lead author (A.L.) developed a draft codebook a priori based on the research questions. Thematic analysis was also used to identify additional themes that emerged from the data, and these additional codes were added into the final codebook. To assess intercoder reliability (ICR), 10% of the transcripts were double-coded by the coding team (A.L. and M.K.S.Q.). The average ICR score was 97%. After coding was completed, outputs for specific codes were generated relevant to service delivery factors that could influence MPT uptake and adherence. These coding outputs were exported to Microsoft Word to enable qualitative analysts at the study sites to participate in the analysis process. Qualitative analysts (A.L., M.K.S.Q., and K.M.) generated summary memos for each code report, and all analysts (A.L., M.K.S.Q., K.M., K.A., J.O., and A.V.D.S.) reviewed the completed batch of summary memos. The qualitative analysis team identified salient themes across the summary memos that were considered factors that would influence delivery, uptake, and adherence to MPTs in these health care settings.

### Ethical Approval and Informed Consent

Written informed consent was obtained from all key informants prior to the interview. Ethical approval for the study was obtained from Pharma-Ethics (South Africa; reference no. 150110905) and KEMRI Scientific and Ethics Review Unit (Kenya; non-SSC protocol no. 474). Key informants received tokens of appreciation worth R35 in South Africa and KES600 in Kenya.

## Results

The majority of key informants were women who, in their role as nurses, offered direct services to a wide range of clients, including minors, adults, and HIV and family planning patients. Other key informants worked in the health care field in a variety of roles such as doctors, medical or clinical officers, counselors, or health care providers in research clinic settings and offered services to the same range of clients as the nurses. On average, the respondents were 37 years old, all but 3 were female, and 79% had a college diploma (Table 2).

**Table 2.** Demographic Characteristics of Health Care Providers.

	IRDO, n = 12	SRC, n = 12	Overall, N = 24
Age	** **	** **	** **
Median age	33	41.5	37
Age range, years	26-53	30-68	26-68
Sex			
Female	75%	100%	88%
Male	25%	0%	12%
Primary Role(s)^a^			
Provide services directly to clients	83%	83%	83%
Supervise staff or other managerial	42%	42%	42%
Job function(s)^a^			
Doctor	17%	8%	13%
Nurse	58%	75%	67%
Counselor	33%	8%	21%
Provider at a research center	0%	8%	4%
Type(s) of clients served^a^			
Adult women	92%	83%	88%
Adult men	92%	58%	75%
HIV/AIDS patients	75%	75%	75%
Family planning clients	100%	83%	92%
Adolescent girls (age 12-18 years)	83%	75%	79%
Adolescent boys (age 12-18 years)	75%	50%	63%
Children	92%	58%	75%

Abbreviations: IRDO, Impact Research & Development Organization; SRC, Setshaba Research Center.

^a^ Multiple responses permitted.

### Perceptions of Young Women’s Interest in the MPTs

Informants thought that most young women will want to try these products, especially if it means they no longer need to use a condom to prevent pregnancy or HIV. Informants highlighted how some of their clients were more concerned about pregnancy than HIV, so they appreciated that these types of MPTs provided, “a method that addresses their primary concern which is pregnancy, while also catered for HIV prevention” (Director of Programs, HIV Programming, Kenya). Informants felt that young women will likely appreciate not having to go to different clinics for their reproductive and sexual health care. Instead, they can get a 2-in-1 product at 1 location, which will save time and limit their interactions with health care providers. In the following conversation, a nurse at a local clinic in South Africa expressed that sentiment:

I: And what product characteristic do you think would be most important to women for a combined HIV and pregnancy prevention?

R: I think time consuming, time management is number one because if I have HIV and contraceptive at the same time I don’t have to go and wait in different queues in the clinic…At least if I go once, and then I see them after 3 months or so, is going to be much better.

Although informants believed that young women will want to use these products, they stressed that women will be concerned about their potential side effects. Routinely mentioned unwanted side effects included weight gain, nausea, decreased libido, impacts on menstruation, and concerns about the effect on future fertility. As a health promoter at a youth clinic in South Africa mentioned, some young women will “be afraid” of these products because they will think “at the end it will make them sterile.” Many of the informants voiced their concerns that if young women are using these products, they will no longer use condoms and providers will have to deal with an increase in other sexually transmitted infections.

Across the 4 products, it was common for the informants to respond that women will like these products because “nobody has to know you have gotten it so you must not necessarily get your partner involved” (Doctor, Public Hospital, Kenya)

### Product Administration

Similar to their perceptions that young women will appreciate the time-saving benefit of MPTs, all of the informants were excited by the possibility of being able to offer young women a product that could “kill 2 birds with 1 stone” (Nurse, Reproductive Health Clinic, South Africa). On the one hand, the informants like that these MPTs could reduce the amount of time required to provide services to young women, but some felt that because “too many people will want [MPTs]” (Nurse, Skills Center at a University, South Africa), it will create more work for providers.

Those interviewed felt strongly that the proposed MPTs would need to be provided by health professionals, including pharmacists, because they will be best equipped to manage any side effects that women may experience while using the products. They felt that in some cases, community health workers could provide the rings or pills and appreciated this, as it would decrease the work burden on providers. However, they emphasized that injections and implants required special training. Therefore, they saw these products being provided primarily within the clinic or pharmacy setting.

Although the informants like the dual prevention aspect of an implant, that women can use it without their partner’s knowledge, and that protection is assured once it is inserted; they did raise concerns about this type of product. Informants talked about the time-intensive nature of inserting and removing the implant. They anticipated that their workload would become overwhelming because so many women would want to use the implant. A nurse at a University Skills Center in South Africa shared that in light of this increased workload, providers would find it easier to “just offer a condom [because] then [it] is easy because you just put them there, then they [take it]” (Nurse, Skills Center at a University, South Africa). Some informants thought that a biodegradable implant would decrease workload because they would not need to do any removals.

Similar to the implant, informants liked the discreetness of this method and that once a woman has received the injection, she is protected against both HIV and unintended pregnancy. A doctor at the Ministry of Health in Kenya commented that “just the same way the Depo-Provera is popular, this one might also be a big one.” Many noted, however, that requiring 2 shots, once a month, is a barrier. They preferred an injection that is longer acting, that only requires a single shot, and that could be synchronized with other clinic services (eg, Depo-Provera shots). Some raised health concerns such as abscesses with frequent injections or other health concerns if injections are given in nonsterile conditions. Although not a majority view, a nurse at a comprehensive care center in Kenya mentioned that in their location injections are considered “tiresome” because of the number of clients they already have to inject. Further she raised the concern of iatrogenic infection, saying: “it is preferred for the clients to use the orals and not injections because they wanted to reduce the HIV transmission rate, and because this will be issued to the HIV negative, the issue of infection prevention by health care providers might also have an issue.”

### Product Attributes

The informants appreciated that all of the proposed MPTs are methods initiated by women. Most of the informants wanted the MPTs to provide a longer duration of coverage, especially those that require clinic administration (injection and implant). This would limit the time burden on the health care providers to dispense these products. The preferred length of duration ranged from 3 months to 3 years. For the pills, some informants requested that women be given a 3- to 6-month supply. Informants highlighted that because of pregnancy desires, younger women will likely prefer a shorter duration of coverage, such as 2 to 3 months when compared to older women who would want coverage for at least 1 year. Informants stressed the importance of the products being easily accessible (both from a provider and from a user standpoint), affordable, and that the product supply be reliable. As a nurse working at a clinic that offers family planning and HIV services reflected (South Africa), “If I don’t get supply and I told people that I have, this is going to be something very bad for me because I promised them that I have this and it is going to work for you…Now I can’t give them what I promised them, is going to impact negatively.”

Many of the informants interviewed highlighted how pill taking is not as discreet as the other options, but it does offer women control over the method. A counselor at a clinic in South Africa commented how men can take condoms off, but with the pill, “You are sure about your story, that this one, I am the one has who drank it.” Even with that benefit, the informants shared a lot of concerns about the pills and their packaging. The primary concerns were that women may be disinclined to use the pills because they look like antiretroviral (ARV) medications used to treat HIV and that the packaging (a standard pill container) also resembles those used for ARV medications. A doctor at the Ministry of Health in Kenya said that, “When I see this bottle, that’s the first thing I remember. They look like ARVs and this blue thing [the pill] looks like one of those.” To overcome this challenge, informants suggested making the pill smaller, changing its color from blue, and using blister packs similar to those used for contraceptives. The additional benefit of changing the packaging to something more discreet and with separate compartments (ie, a blister pack) for each pill is that it would allow women to take it with them and would prevent pills from spilling out and getting lost. Pills compartmentalized by day may also help with adherence. Informants highlighted the anticipated challenge of women forgetting to take their pills daily which would decrease protection.

The informants felt that as long as sex partners cannot feel the ring, no one will know that women are using it. Some reflected that the ring looked hygienic and easy to use. However, they highlighted several attributes they felt would prevent women from wanting to use it. They worried that it was too hard and would be painful to the woman using it. As one counselor at a clinic in South Africa reflected, “The vagina is a sensitive thing.” Some suggested the ring be modified to be more of a sponge-like texture and that this may also prevent male partners from feeling or being hurt by it during vaginal sex. Informants in Kenya shared that hygiene and sanitation may also be a barrier for ring use. A doctor at a public hospital in Kenya highlighted how with the ring, “you need to basically live in a place where you can get water easily to wash your hands and that is not true for our setup. Some—most of our ladies do not have access to easy and clean water even for drinking.” Similar to the pills, informants were concerned about adherence issues because women can take the ring out when they want. Although most informants felt that the packaging of the ring was acceptable, some suggested using more “feminine colors” (Nurse, clinic, South Africa), decreasing the size and noisiness of the packaging, and making one side of the packaging transparent so providers can show the ring to women without having to open the packaging.

Informants raised concerns about the biodegradable feature of an implant. For some, the key issue stressed was that biodegradable implants must be removable in case someone experiences problematic side effects. They also noted the importance of educating young women to feel comfortable about using such a product because they will be concerned about where the implant goes in their bodies after it disintegrates.

### Providers’ Training Needs

Consistently, informants stressed the importance of receiving training prior to offering these MPTs. Training needs mentioned included a thorough understanding of how the products work, their efficacy levels, potential side effects, reversibility, and for new delivery forms like the vaginal ring, how to insert it. A nurse at a youth center clinic in South Africa spoke to the importance of having trained providers:

[T]he person who is giving it to them it [must] be somebody that can be approached, having an insight and light, having information about the method, must not be like “let me go and ask first how does this thing work.” They must be trained on how to give this so that they can give the client the correct information because if you don’t know it yourself how do you give to the next person who’s not informed about it.

Ultimately, if you do not have well-informed, trained providers, they will not promote the products or provide accurate information about them.

Not surprising, the one delivery form that was new to most of the respondents was the vaginal ring. The novelty, size, and need for vaginal insertion prompted informants to express many of the same concerns that women in ring studies voiced prior to trying the product.^23,24^ Their “fear of the unknown” (Nurse, Family Planning Clinic, South Africa) included whether it would be painful to insert, if a male partner would feel it or be hurt by it during sex, and negative side effects from leaving a foreign object in the vagina for a long period of time. After first seeing the ring, a female counselor at a clinic in South Africa responded, “I won’t put that thing inside of me” (Counselor, Clinic, South Africa). Informants acknowledged that if their fears were unfounded, the ring would be a great product because “You put it and you stay 1 full month without removing it and you are just doing your errands. You are not afraid of sex because there is something inside you that was put there. You don’t need to worry about safe sex or prevention” (Nurse/Counselor, County Hospital, Kenya). These concerns highlight the importance of product-specific education, especially for those products previously unknown to providers.

## Discussion

The health care providers in this study expressed great initial interest toward MPTs and some excitement at the possibility of offering MPTs to their clients; they also anticipated that the majority of their clients would be interested in using them. The only noticeable difference between the 2 sites was that informants in Kenya raised concerns about hygiene and sanitation issues that may impede ring or injection use. Aside from that one country-specific concern, key informants uniformly expressed a clear interest in seeing new MPT products become available, especially those that are longer acting (injection, ring, and implant).

Most key informants highlighted the adherence issues young women already experienced taking contraceptive pills and as a result preferred the other methods that did not require daily action on the part of the user. Similarly, a study with staff and clients of facilities providing contraception in the United States found that the “forgettable” nature of IUDs and implants provided a benefit over more temporary contraceptive methods—in this study, this finding was even more pronounced among the clients than the facility staff.^25^ In this study of providers in South Africa and Kenya, most informants advocated for products that offered a longer duration of coverage, but their preferred duration reflected their perceptions about women’s pregnancy desires. For women who may want to get pregnant, informants advocated for a product that would offer 2 to 3 months of coverage. For those women who are not looking to get pregnant, they advocated for a product that would provide at least 1 year’s worth of coverage. This seems to mirror findings from a study conducted with health care providers in Uganda about providing contraceptive services to young women.^26^ In that study, the providers voiced their preference for short-acting contraceptive methods for young married women, although the majority of the providers interviewed did not believe that contraceptives should be provided to young women due to potential long-term side effects or finding it to be “morally unacceptable.”

Although the key informants expressed interest in supplying or providing MPTs, they highlighted some practical challenges at the clinic level that need to be addressed. Several overarching themes illustrate these potential resource challenges: time, consistent stock supply, inventory controls, and staffing needs. Time constraints related to counseling and education have been noted in other studies exploring health care providers’ perspectives about contraception.^25,27^ Across the sites in this study, health system improvements named included inventory controls, adequate staffing to meet the demand, and consistent stock supplies. Consistent stock supply was raised as a concern in another study and reflects some of the unique constraints of resource-limited countries.^26^

Health care providers will be the gatekeepers for these MPTs, and if they do not have a clear understanding of the products (ie, how they work, potential side effects, duration of coverage, and reversibility) and an ability to share this information in a way that is accessible for young women, the uptake of these products will be compromised. The informants interviewed recognized their biases and the greater concerns they had about technologies unknown to them, such as the ring or biodegradable implant. Regardless of the product type, providers consistently highlighted the importance of receiving adequate training about the proposed MPTs. This need has been raised by others in connection to pre-exposure prophylaxis.^28-30^ Recognizing how many of the key informants raised concerns that uptake of MPTs may cause an increase in sexually transmitted infections (STIs), trainings will also need to offer guidance on how to talk to MPT users about the lack of STI protection conferred, routine testing for STIs, and condom negotiation.

### Strengths and Limitations

This study is the first to document health care providers’ perspectives on the acceptability and feasibility of offering MPTs to young women in South Africa and Kenya. These data have several limitations. Although sites recruited a diverse sample of health care providers in each location, this is a small qualitative nonrandom sample; thus, findings may not be generalizable to other health care providers within and outside these locations. Additionally, all informants were interviewed in English. Potential informants were screened for comfort being interviewed in English; thus, all informants’ knowledge of English was sufficient to collect high-quality data in our interviews. However, it is possible that selection of informants who are proficient in English may have biased our sample. However, the input of these health care providers is useful in considering the support needed for future implementation of MPTs. Those interviewed were prompted to assume that the proposed products had equal efficacy levels. Had specific efficacy levels been presented for the placebo products, informants’ responses likely would have changed. The intent of this study was to assess health care providers’ responses to these specific potential MPT’s attributes and administration techniques.

The responses of this sample of health care providers complement the perspectives of young women, as evidenced by research done with women, which focused on potential users’ perspectives and other components of this same study. Previous and concurrent research into young women’s preferences for HIV prevention products have shown that women have strong preferences for products that last longer, are easy to use, and have minimal user burden for administration^15,31,32^ but nevertheless value choice and thus multiple options for prevention.^33^ Additionally, quantitative results showed that women view product efficacy as the most important attribute of these products, followed by an MPT indication.^34^ In this study, too, informants mostly prioritized effectiveness as well as safety and reversibility. In addition, they emphasized considerations about provider burden and the health care system’s capacity. Importantly, they indicated that younger women would want products that provide shorter durations of protection, as they may have changing fertility desires. In recognition of a potential for misalignment between user preferences and providers’ priorities, this sample of key informants stressed the importance of ensuring end-users’ voices inform the development of new health technologies.

A subsequent component of this study provided young women with placebo versions of the ring, injection, and pills to try for at least 1 month each,^17^ and these women provided feedback both quantitatively and qualitatively. Full analyses of women’s perspectives on these products have been published elsewhere using quantitative and qualitative responses.^19,33,18^ An analysis of perspectives from male partners of study participants is forthcoming. Given all the above-mentioned limitations, the feasibility of providing these MPTs warrants further consideration in health care settings throughout sub-Saharan Africa.

Recognizing the important role that health care providers play in delivering new technologies, this study provides concrete examples of the product features and attributes that health care providers are interested in offering and also consideration about the infrastructure required for successful delivery. It also suggests that if time constraints, staffing needs, training, and stock supply barriers are minimized, health care providers are eager to be able to offer a product that prevents both HIV and unintended pregnancy to young women in their settings.
